# Transformer hot spot temperature estimation through adaptive neuro fuzzy inference system approach

**DOI:** 10.1016/j.heliyon.2024.e26338

**Published:** 2024-02-13

**Authors:** Edwell T. Mharakurwa, Dorothy W. Gicheru

**Affiliations:** Department of Electrical & Electronic Engineering Dedan Kimathi University of Technology (DeKUT), Private Bag, 10 143, Nyeri, Kenya

**Keywords:** Hot spot temperature, Top oil temperature, Adaptive neuro-fuzzy inference system, Dynamic loading, Power transformer, Soft computing, Thermal model

## Abstract

Transformer performance and efficiency can be enhanced by effectively address the properties of its insulation system. The power transformer insulation system weakens as a result of operational thermal stresses brought on by dynamic loading and shifting environmental patterns. Winding hot spot temperature is a crucial metric that must be maintained below the prescribed limit while power transformers are operating so as to maintained power system reliability. This is due to the fact that, among other variables, the time-dependent aging effect of insulation depends on transitions in hot spot temperatures. Due to the non‐linear nature of the conventional mathematical models used to determine these temperatures, and complexity of thermal phenomena, investigations still need to be exercised to fully understand the variables that associate with hot spot temperature computation with minimum error. This paper explores the possibilities of enhancing top oil and hot spot temperature estimation accuracy through the use of an adaptive neuro-fuzzy inference (ANFIS) technique. The paper presents an adaptive neuro fuzzy model to approximate the hot spot temperature of a mineral oil-filled power transformer based on loading, and established top oil temperature. Initially, a sub-ANFIS top oil temperature estimation model based on loading and ambient temperature as inputs is established. Using a hybrid optimization technique, the ANFIS membership functions were fine-tuned throughout the training process to reduce the difference between the actual and anticipated outcomes. The correctness and reliability of the created adaptive neural fuzzy model have been verified using real-world field data from a 60/90MVA, 132 kV power transformers under dynamic operating regimes. The ANFIS model results were validated against field measured values and literature-based electrical-thermal analogous models, establishing a precise input-output correlation. The developed ANFIS model achieves the highest coefficient of determination for both TOT and HST (0.98 and 0.96) and the lowest mean square error (7.8 and 10.3) among the compared thermal models. Correct determination of HST can help asset managers in thermal analysis trending of the in-service transformers, helping them to make proper loading recommendations for safeguarding the asset.

## 1 Introduction

Power transformers are among the most capital-intensive assets of the electrical power system and their life management scheme is vital for the continuous and reliable operational of the power grid. Due to increase demand for energy in different sectors of the world and load growth, power system assets in particular transformers, are being stretched to the limits leading to accelerated deterioration to their conditions. Stressing a power transformer for a long period can lead to untimely faulting or failure, thus, interrupting continuous power flow to consumers. However, addressing transformer life threatening variables through the timely condition based or scheduled maintenance can improve the remnant life span of a power transformer. In literature, it is reported that typical transformer faulting likelihood is linked to the transformer load-ability regime [1]. Load increase has contributed to the continuous rise of the transformer's maximum temperature in which the windings and the insulation system suffers. These temperatures are normally referred to as the hot spot temperature (HST). There is a direct correlation amongst HST, the deterioration of the transformer insulation and to the faulting probability of the transformer's components [[2], [3], [4]]. Thus, monitoring, predicting and controlling HSTs under dynamic loading conditions and different ambient temperatures enable the prevention of the transformer's untimely thermal incipient faults, thereby enhancing longevity of power transformers technical life.

The power transformer loading capacity is vividly associated with its variant thermal conditions, which can be projected using dynamic thermal models. As highlighted in different manuscripts, thermal models illuminated the intricate heat transfer phenomena within the transformer in the form of simplified differential equations with a notion of representing primarily the top oil temperature (TOT) and the HST [5]. Winding hot spot temperature is directly corelated with the technical lifespan of the power transformer via the thermal stress imparted to the insulation system. It is therefore of paramount importance to correctly measure and control this parameter. It is observed that use of optical fiber sensors in the linings of phase windings can accurately determine the thermal hot spots of the transformer windings [[6], [7], [8], [9], [10]]. However, this technique is capital intensive and also not practically applicable to in-service and aged transformers without retiring them from operation which may disrupt the continuous supply of power. This has technically enforced continued adoption of winding temperature indicators (WTI) as the hot spot temperature measurement practice in aged and some new transformers. Consequently, different thermal models have been formulated so as to aid in the estimation of transformer TOT and HST. In Ref. [3], it is reported that thermal models can be grouped as physical models, semi-physical models or computational fluid dynamic based models.

Findings reported in literature have contributed immensely in improving the accuracy of dynamic models by considering different variables associated with thermal behavior of power transformers. The environmental aspect in the form of wind-speed, and its direction was considered in improving the estimation accuracy of the thermal model as reported in Ref. [11]. Additionally, the solar radiation intensity which was overlooked in the IEC and IEEE standardized loading guides [12,13] in determining transformer temperature changes was considered in models developed in Refs. [[14], [15], [16], [17]]. It was further confirmed through applications that solar radiation can lead to transformer top oil temperature rise by about 3.7 °C, [17]. Other variables considered by different researchers in thermal dynamic model modifications for TOT and HST estimation to improve on adequacy and accuracy are not limited to tap-changer position, oil viscosity, harmonics in loading, insulation water content, constant or variable thermal resistances taking into account the type, design and capacity of the transformer [5,[16], [17], [18], [19], [20], [21]].

As the data related to the transformer thermal behavior are progressively accessible, it is possible to formulate a transformer thermal model based on soft computing techniques regardless of thermal attributes. Power transformer estimation and prediction thermal models based on the available data were developed through exploitation of artificial neural network (ANN) and the obtained results were superior to the one obtained through conventional approaches, but well comparable to the practical-measured data [[22], [23], [24], [25]]. In addition, the support vector machine (SVM) [26], fuzzy [27,28] fuzzy-NN [29], were investigated in estimating the hot-spot temperature for a power transformer. Nevertheless, long-term performance of the artificial intelligent-based approaches, particularly ANN, over the years, and across different assets, were not adopted due to inaccessibility of field measurement data. However, harnessing the advantages brought by the aspects of soft computing techniques and availability of data will enhance the precision and accuracy of thermal models for hot spot temperature estimation and prediction. The principal drive in transformer thermal modeling through TOT and HST forecasting is in establishing measures to extend the technical calendar life duration of the in-service transformer insulation system subjected to thermal stresses. Though there have been innovative contributions for estimating the hot spot temperatures centered on modified thermo-electric analogy methods or hybridized methodologies, it is necessary to investigate these methods for more accurate and effective HST valuation models.

Given the difficulty in creating highly accurate deterministic models, a number of recent publications have used black box models to perform thermal modeling that are developed using historical data. History-based techniques such as neural networks and fuzzy systems, which can handle uncertain data and learn complex nonlinear relationships, have been demonstrated to be effective in thermal modeling. However, each of these individual models is missing a crucial element, "adaptability', that would limit their applicability to evolving transformer thermal behavior. It is anticipated that the behavior of the transformer will evolve as a result of variations in external inputs, structural modifications, different maintenance strategies, and other factors. Therefore, if the attributes or the structure of the model do not adapt to account for these changes over time, the model may become invalid.

This paper suggests the use of the ANFIS model to perform thermal modeling of power transformers. The objective is to predict the transformer winding hot spot temperature using current and past data. The main contribution of the present work focuses on the efficacious optimization, validation, and development of intelligent ANFIS model that estimate the power transformer TOT and HST. The benefit of this model is its adaptability to system changes, which makes it a competitive choice for use in practical situations where the behavior of the transformer performance may vary over time. This paper further explores on the accuracy and adequacy of the transformer thermal model (TOT and HST estimation) by harnessing the merits of the Adaptive Neuro Fuzzy Inference System (ANFIS) for transformer thermal condition valuation. The variables adopted in the formulation of this model are the dynamic transformer loading regime, environmental parameters in the form of ambient temperature. A significant forte of the proposed thermal estimation model in this study is the adoption of the entwined effect of these two often measured attributes for top oil temperature estimation. Thus, a soft computing intelligent strategy that considers the adaptive fine-tuning of rules formulated on gathered data and knowledge of the actual measurements of the parameters involved is utilized in decision mapping of the outcome. An additional noteworthy implication of this study is the potential utilization of the ANFIS models as a robust alternative to conventional mathematical thermal tools in the context of power transformer thermal analysis.

The remaining sections of the paper is structured follows; Section 2 details the brief literature on conventional methodologies employed in transformer thermal analysis. Section 3 describes some of the established soft computing techniques in transformer thermal analysis. Section 4 articulates the procedure in establishing the ANFIS transformer thermal model. Section 5 addresses the simulation results and comparisons. Finally, the conclusions and further prospective developments are summarized in Section 6.

## 2 Brief review of conventional transformer thermal analysis

Thermal stress, which is primarily influenced by loading circumstances and ambient temperature, has a direct impact on how well a transformer's insulation holds up over time. One of the most crucial components for determining the state of a transformer is a thorough analysis of transformer thermal behavior in the form of hot spot temperatures. To offer direction on identifying suitable ratings and operation modes for transformers, many transformer loading recommendations have been presented. The popularly adopted conventional thermal models for estimating transformer hot spot temperatures are briefly discussed. Although many thermal model improvements have emerged, most of them rely on the foundation of models outlined by the IEEE model, Swift's model and Susa's models which are discussed in this section.

### 2.1 IEEE models

As estimated by IEEE loading guides [13], oil and winding temperature rise can be due to rise in loading regimes that activates the increase in winding currents. The top oil temperature rise is calculated as per expressions (1) and (2) which resembles an exponential response behavior [13].

(1) $$\tau_{o,R}\frac{{d\Delta \theta }_{o}}{dt}={\Delta \theta }_{o,U}-{\Delta \theta }_{o,I}$$

(2) $${\Delta \theta }_{o,U}={\Delta \theta }_{o,R}.\left(\frac{{1+RK}^{2}}{1+R}\right)$$

where ${\Delta \theta }_{o,I}$ is the initial top oil temperature rise, ${\Delta \theta }_{o,U}$ represents the ultimate top oil temperature rise, $\tau_{o,R}$ denotes rated oil time constant, ${\Delta \theta }_{o}$ represents the top oil temperature rise, *R* shows the ratio between transformer load loss and loss of transformer during no load test, *K* denotes the ratio between load current and rated winding current.

Similar to top oil temperature calculations, the exponential response from the initial hot spot temperature-rise over top oil temperature $\left({\Delta \theta }_{hs,I}\right)$ to the ultimate hot spot temperature rise over the top oil temperature (${\Delta \theta }_{hs,U}$) is employed in the transformer hot spot temperature rise calculations as highlighted in Ref. [13] by expressions (3) and (4):

(3) $$\tau_{wd,R}\frac{{d\Delta \theta }_{hs}}{dt}={\Delta \theta }_{hs,U}-{\Delta \theta }_{hs,I}$$

(4) $${\Delta \theta }_{hs,U}={\Delta \theta }_{hs,R}.\left(K^{2m}\right)$$

where, $\tau_{wd,R}$ is the rated winding temperature time constant and m is a constant for the nonlinearity of hot spot thermal resistance for different cooling modes. After some mathematical formulations, the final HST equation is formulated as a combination of variables highlighted in expression (5) [13],:

(5) $$\theta_{hs}=\theta_{a}+{\Delta \theta }_{o}+{\Delta \theta }_{hs}$$

where, $\theta_{hs}$ shows hot spot temperature, the hot spot temperature rise is noted by ${\Delta \theta }_{hs}$ and $\theta_{a}$ symbolizes environmental temperature. Through further study, Lesieutre et al. [30], updated the IEEE models as in (6), (7).

(6) $$\tau_{o,R}\frac{{d\Delta \theta }_{o}}{dt}={\Delta \theta }_{o,U}+\theta_{a}-{\Delta \theta }_{o,I}$$

(7) $$\theta_{hs}=\theta_{o}+{\Delta \theta }_{hs}$$

### 2.2 Swift's model

Centered on heat transfer theory, Swift et al. [31,32], formulated an equivalent circuit for the transformer thermal model. In this model, the top oil and HST were determined by considering mineral oil nonlinear thermal resistance. The final dynamic thermal expressions for TOT and HST formulated from the equivalent circuit are stated as in (8), (9).

(8) $$\frac{K^{2}R+1}{R+1}.{\Delta \theta }_{o,R}^{\frac{1}{n}}=\tau_{o,R}\frac{{d\theta }_{o}}{dt}+{\left(\theta_{o}-\theta_{a}\right)}^{\frac{1}{n}}$$

(9) $$K^{2}{\Delta \theta }_{hs,R}^{\frac{1}{m}}=\tau_{wd,R}\frac{{d\theta }_{hs}}{dt}+{\left(\theta_{hs}-\theta_{o}\right)}^{\frac{1}{m}}$$

where, n and m are constant signifying nonlinearity in oil thermal resistance for diverse cooling modes.

### 2.3 Susa's model

Susa upgraded Swift's model by incorporating the non-linear thermal resistance of mineral oil which depends also on temperature, oil viscosity and loss variation [19,33,34]. Susa's computational top oil temperature model is denoted by (10):

(10) $$\frac{K^{2}R+1}{R+1}.{\Delta \theta }_{o,R}=\tau_{o,R}\frac{{d\theta }_{o}}{dt}+{\frac{\left(\theta_{o}-\theta_{a}\right)}{{\Delta \theta }_{o,R}^{n}.\mu_{pu}^{n}}}^{1+n}$$

where, $\mu_{pu}$ represents the ratio of oil viscosity between current measured temperature and rated top oil temperature. Susa's final hot spot temperature thermal equation is expressed as in (11):

(11) $$K^{2}.{P_{Cu.pu}.\Delta \theta }_{o,R}=\tau_{wd,R}\frac{{d\theta }_{hs}}{dt}+{\frac{\left(\theta_{hs}-\theta_{o}\right)}{{\Delta \theta }_{hs,R}^{m}.\mu_{pu}^{m}}}^{1+m}$$

where, $P_{Cu.pu}$ signifies the transformer winding copper losses values in per unit.

To take into account the effect of water content in the solid insulation in temperature variations on transformer thermal behavior, Cui et al. [20], updated Susa's model and the resultant models are demonstrated in equations (12), (13). In Ref. [16], the aspect of the solar irradiation and moisture effect on the top oil temperature was taken into account and the resulting model is articulated in expression (14)

(12) $$\;\;\;\left[\frac{K^{2}.R+1}{R+1}\right].{\Delta \theta }_{o,R}\;=\;\tau_{o,R}.\frac{d\theta_{o}}{dt}+\left[\frac{{\left(\theta_{o}-\theta_{a}\right)}^{n+1}}{{\Delta \theta }_{o,R}^{n}}.\frac{1+\eta_{R}}{\mu_{pu}^{n}+\eta_{R}.r_{pu}{\left[\frac{{\Delta \theta }_{o}}{{\Delta \theta }_{o,R}}\right]}^{n}}\right]$$

(13) $$K^{2}P_{Wnd,pu}.{\Delta \theta }_{hs,R}=\tau_{wd,R}.\frac{d\theta_{hs}}{dt}\left[\frac{{\left(\theta_{hs}-\theta_{o}\right)}^{m+1}}{{\Delta \theta }_{hs,R}^{m}}.\frac{1+\eta_{R}}{\mu_{pu}^{m}+\eta_{R}.r_{pu}{\left[\frac{{\Delta \theta }_{hs}}{{\Delta \theta }_{hs,R}}\right]}^{m}}\right]$$

(14) $$\left[\left[\frac{K^{2}.R+1}{R+1}\right]+\frac{q_{Rad}}{q_{Fe}+q_{Cu}}\right].{\Delta \theta }_{o,R}=\tau_{o,R}.\frac{d\theta_{o}}{dt}\left[\frac{{\left(\theta_{o}-\theta_{a}\right)}^{n+1}}{{\Delta \theta }_{o,R}^{n}}.\frac{1+\eta_{R}}{\mu_{pu}^{n}+\eta_{R}.r_{pu}{\left[\frac{{\Delta \theta }_{o}}{{\Delta \theta }_{o,R}}\right]}^{n}}\right]$$

where, $r_{pu}$ denotes the ratio of cellulose thermal resistance between any temperature and rated temperature, $\eta_{R}$ shows the ratio of rated thermal resistance of solid insulation (cellulose) and rated thermal resistance of liquid insulation (oil).

## 3 Brief review of soft computing techniques in transformer thermal analysis

The possibility of a valid and accurate "one size fits all" mathematical model is unlikely given the complexity and variability in transformer design and operation. Also, some other factors for the implementation of the aforementioned thermal dynamic models may need to be acquired beforehand from the manufacturers, like “heat run test" [26]. These parameters might not be readily accessible, notably for those aged transformers. Statistical machine learning methods are the foundation of an innovative hot spot temperature measurement strategy [35]. With the aid of a historical dataset for a transformer (such as hot spot temperature, TOT, load current, ambient temperature, bottom oil temperature, etc.), soft computing techniques such as ANN and SVR are able to recognize the intricate and nonlinear patterns in the time series of transformer thermal profile and predict hot spot temperature over a given period of time.

Several researchers are focusing their studies on the application of artificial intelligence algorithms to predict the HST in accordance with the transformer operation data in order to quickly and effectively estimate the transformer winding HST. The benefit of using an artificial intelligence-based algorithm to determine HST in a transformer is that the trained model can be used to estimate HST in an operating transformer while taking into account variables like the transformer's structure, the material's non-uniformity, and the impact of environmental conditions. This can improve the utilization of the transformer's capacity while maintaining its dependability. Additionally, a comparison of the hot spot temperature between the expected and actual measurements may reveal any anomalies in the transformers. In Refs. [[36], [37], [38]], transformer top oil temperature estimation was achieved through the use of ANN, in which the ambient temperature and the load current were mainly employed as inputs.

Using a radial basis function neural network (RBFN), Galdi and Ippolito were able to accurately detect the HST of a 25kVA distribution transformer than the IEEE empirical calculation [39]. The results of Q. He's [22] discussion of the viability of three different neural networks for transformer TOT detection demonstrate that temporal processing networks can produce the most precise predictions. The HST transformer was also detected using a fuzzy neural system, which has superior accuracy and computational efficiency compared to RBFN and multilayer neural networks [40,41]. Villacci [42] used the idea of grey systems in transformer HST valuation and realized that the IEEE empirical formula and machine learning technique can be cascaded to improve on model accuracy. The performance of three widely used models (IEEE model, Swift's model, and Susa's model) for forecasting transformer top oil temperature is compared in Ref. [43] on the basis of support vector regression optimized using genetic algorithm (GA).

In addition to the aforementioned intelligent analysis technique, the HST pattern of the transformer can also be achieved by employing the support vector machine model. Estimation of the HST of an oil submerged transformer, was established via a support vector regression (SVR) with gradient descent optimization approach as highlighted in Ref. [44]. Additionally, in Ref. [45] a 750MVA/500 kV power transformer's HST was found using the SVR approach, and the method had good detection success. Cui [26], suggested a SVR method that uses information granulation to predict the HST of a 15MVA, Oil Directed Air Forced Cooling (ODAF) transformer and this approach surpasses several already adopted thermal model-based techniques. With effective parameter optimization of the SVR model, Deng et al. [46], managed to predict HST for a 100kVA/10 kV oil submerged transformer. The model has a maximum temperature difference of 3 °C after comparing with the field obtained data, showing the viability of the established model. An inversion detection technique for transformer transient hot spot temperature was established in Ref. [47]. The results of this methodology outperform the outcome of GA-BPNN method in HST determination. The aforementioned techniques demonstrate the efficacy of an AI system in the computation of transformer winding HST, nevertheless, the transformer thermal behavior and modeling has not yet been exhaustively explored.

### 3.1 Transformer thermal model

Mathematical models have been accepted in the estimation of transformers hottest point; however, they also have their limitations. The adaptive neuro fuzzy inference system (ANFIS) has emerged to be an alluring, powerful, general modelling tool, compounding well established learning principles of ANNs and the linguistic transparency of fuzzy logic theory. Thus, the ANFIS is given the learning ability from training data in the same way that an ANN does by using the ANN technique to update the Takagi-Sugeno type inference model's parameters. As a result, the outcome that have been mapped into a fuzzy inference system (FIS) can be expressed using linguistic labels (fuzzy sets). In the ANFIS network, a FIS can therefore exactly determine the nodes and the hidden layers. This improves the prediction ability of ANN models while also minimizing challenge of detecting the hidden layer.

**Fig. 1:**
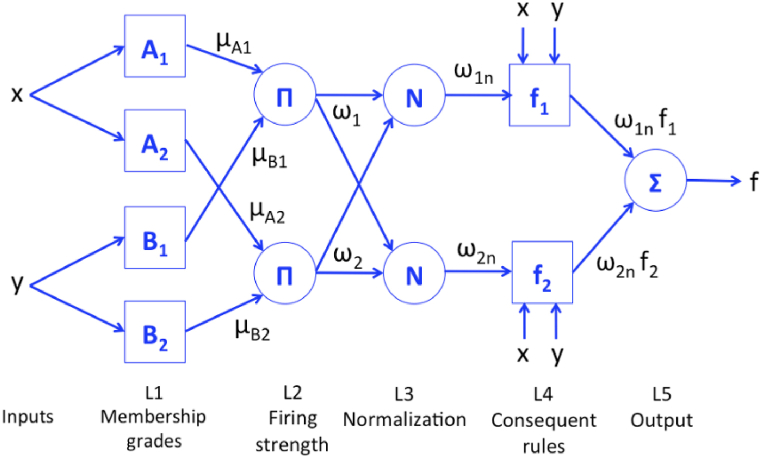
ANFIS topology.

To further explore the possibilities of enhancing accuracy in transformer thermal estimation, this article presents a non-intrusive soft computing method based on the adaptive neuro fuzzy inference system to map the HST of a power transformer centered on routinely measured parameters. By harnessing the unique performance of neural networks and fuzzy inference system, the ANFIS technique is able to explicitly learn from the expert knowledge, thereby enabling it to enhance its performance in addressing nonlinear and complex problems. As discussed in Refs. [48,49], the formulation of a fuzzy inference system relies on the natural language rule formulation established as per expert's interpretation from provided data. Additionally, unless modified by the user, partitioned ranges and the allotted membership functions shape remain fixed. By modifying their parameters in response to any change in the input/output data, ANFIS enables the adaptive fine-tuning of the membership functions [49]. As a result, the ANFIS responds quickly and can adjust to changing data and learn from it. Additionally, ANFIS is taken into consideration since it doesn't need a complicated mathematical model, it is quick and adaptive, and the generated prediction tool can be deployed quickly, which is crucial for estimating transformer thermal profile. A characteristic ANFIS structure is depicted in Fig. 1 [49].

**Layer 1**: Membership grades (ex. Gaussian)

$$<listaend>O_{{L1A}_{i}}=\mu_{A_{i}}\left(x\right)=\exp\;\left[{\left(\frac{x-c_{A_{i}}}{\sigma_{A_{i}}}\right)}^{2}\right]$$

**Layer 2**: Firing strengths

$$O_{L2,i}=\omega_{i}=\mu_{A_{i}}\left(x\right)\times \mu_{B_{i}}\left(y\right),i=1,2$$

**Layer 3**: Normalized firing strengths

$$O_{L3,i}=\omega_{in}=\frac{\omega_{i}}{\omega_{1}+\omega_{2}},i=1,2$$

**Layer 4**: Consequent rules

$$O_{L4,i}=\omega_{in}\times f_{i}\left(x,y\right),i=1,2$$

**Layer 5**: ANFIS output

$$O_{L5}=\sum_{i}^{2}\omega_{in}\times f_{i}\left(x,y\right)$$

**Fig. 2:**
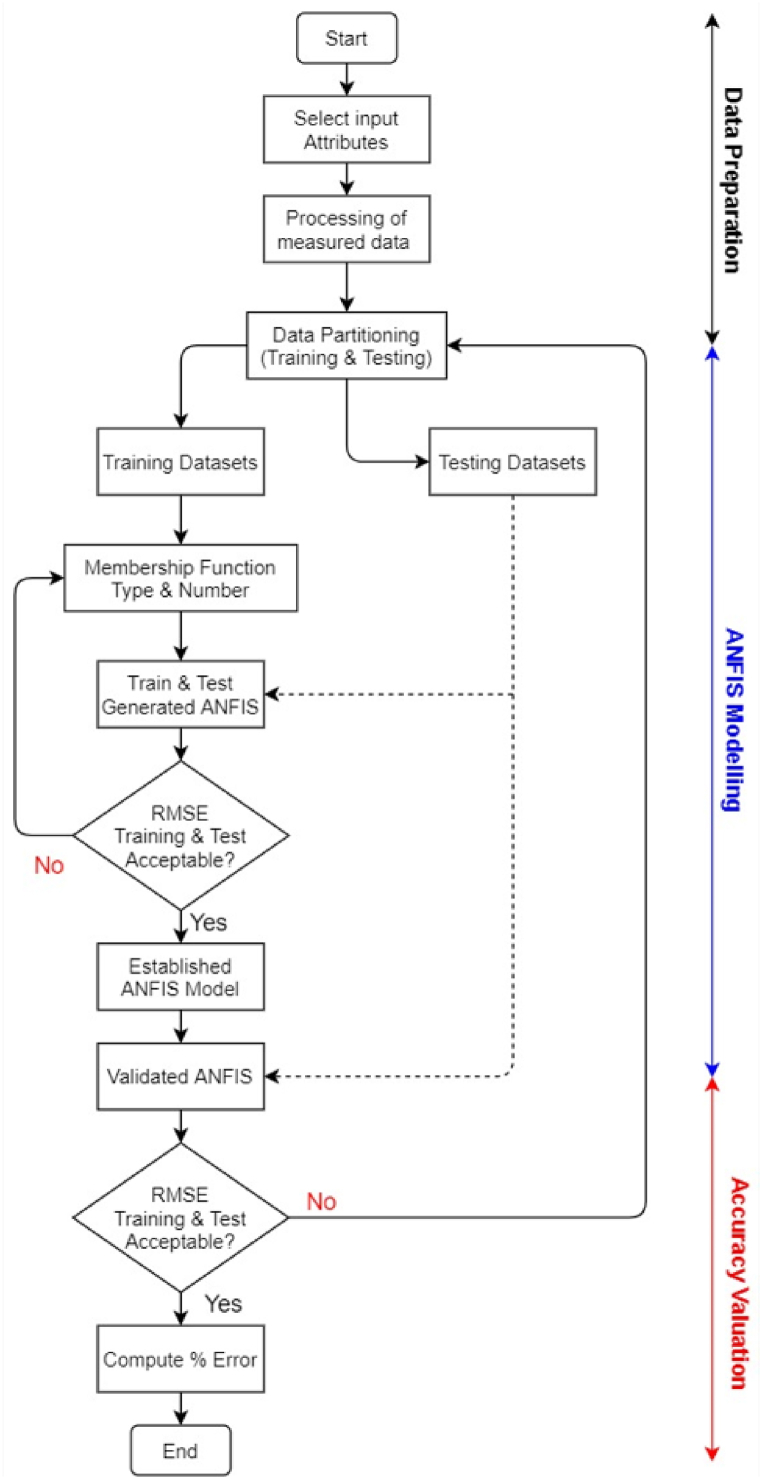
ANFIS flowchart for modelling transformer temperature estimation.

In determining the HST, firstly a sub-ANFIS model for the top oil temperature was realized by keying load profile data and the measured ambient temperature for various transformers operating subjected to diverse environments which are then mapped into an output of top oil temperature value. Field data consisting of the loading profile (p.u), ambient temperature and TOT from installed temperature indicators obtained from at least 5 transformers for a continuously hourly period for at least three (3) months for each transformer was divided into data sets, 70% for training and 30% for testing. Using a hybrid optimization technique, the ANFIS membership functions were fine-tuned throughout the training process to reduce the difference between the actual and anticipated outcomes. However, the results presented in this paper are based on one of the case study transformers (60/90MVA, 132 kV). The proposed procedure for building an ANFIS model for transformer top oil and hot spot temperature estimation is highlighted in Fig. 2.

The procedure for ANFIS modelling for transformer temperature estimation involves three primary stages as noted below:

[1] **Data Preparation:** This involves preparation and processing of data that links to transformer TOT and hot HST. The data analyzed include loading profile, measured ambient, TOT and HST. The data attained from at least 5 transformers for a period of 3 months are then divided into training and testing datasets, 70% datasets used for training, and 30% datasets used to evaluate the developed model.
[2] **ANFIS Modelling:** The ANFIS model is constructed using the training dataset. Finding the ideal combination of Membership Function is the following stage (MF). The input's MF number and type are chosen, the training dataset is employed to train each combination's model, and then each model is assessed to determine which combination works best.
[3] **Accuracy Valuation:** The proposed model is evaluated with the testing datasets, and the error from both training and testing is computed.

**Fig. 3:**
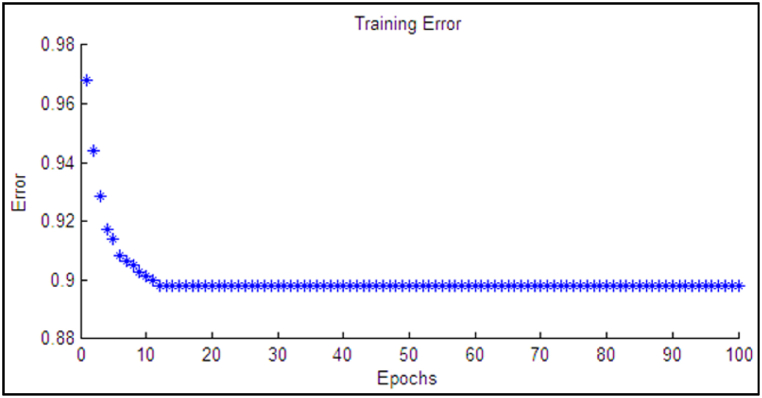
Training error.

The deviation between the expected outcome (TOT) and the measured temperature in the form of the training error is depicted in Fig. 3.

**Fig. 4:**
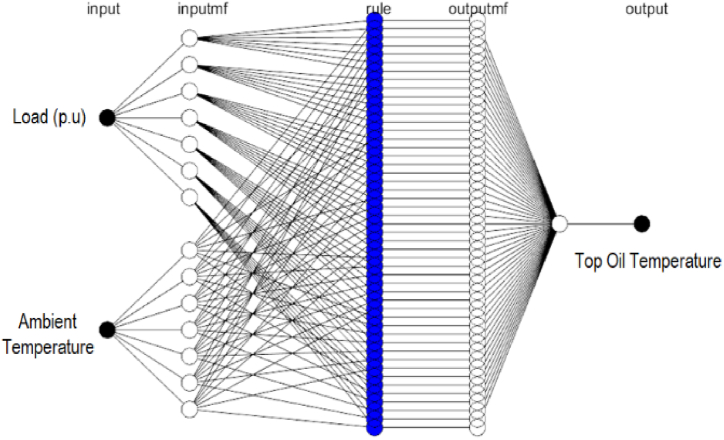
Generated ANFIS structure.

**Fig. 5:**
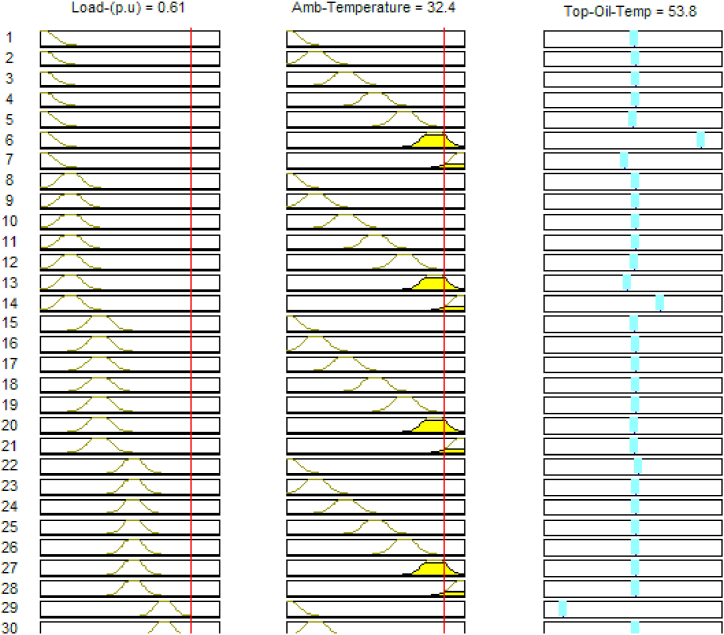
Rules for the established TOT ANFIS model.

The structure of the created ANFIS model is depicted in Fig. 4. The model's utilized input elements are the transformer loading regime (p.u) and the magnitude of the measured ambient temperature, and the expected output variable is the projected top oil temperature value. In Fig. 5, the populated rules allied with the established ANFIS model are represented.

**Fig. 6:**
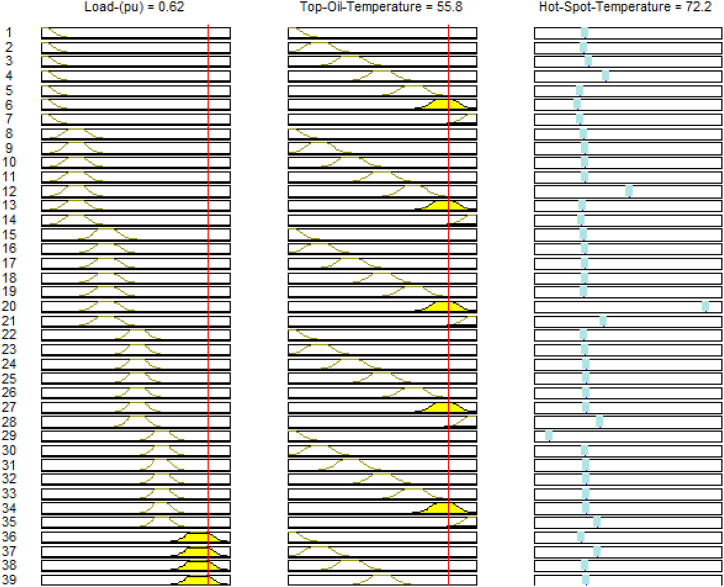
Rules for the established HST ANFIS model.

**Fig. 7:**
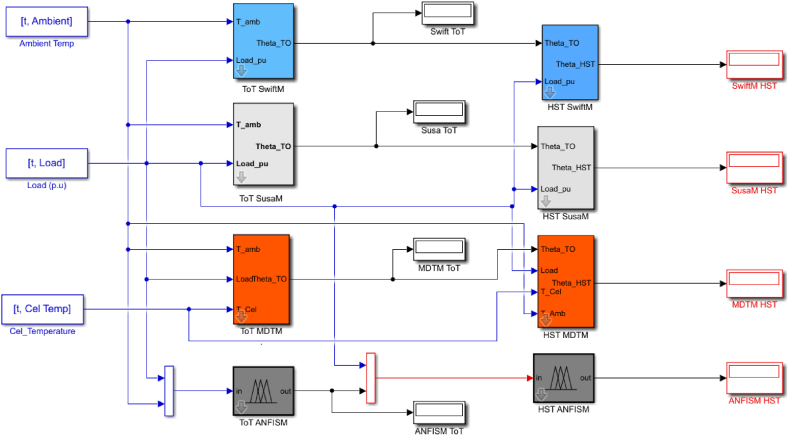
The Simulink model for HST.

Analogous to the formulation concept for the top oil temperature model, the ANFIS model for hot spot temperature consists of loading profile and TOT values as inputs. The TOT values used were those of the ANFIS sub model for top oil temperature. In Fig. 6 the populated rules associated with the developed HST ANFIS models are graphically represented. The SIMULINK sub-models for the developed Adaptive Neuro Fuzzy Inference system and the other conventional models for transformer top oil and hot spot temperatures are portrayed in Fig. 7.

### 3.2 Performance evaluation

Through computation of adequacy and accuracy metrics [50] for the estimated TOT and HST, with reference to on-site measurements, three traditional thermal models and the proposed thermal models were statistically compared. Equation (15) illustrates the coefficient of determination (R^2^) to determine sufficiency, and equation (16) represents the mean squared error (MSE) to determine accuracy and expression (17) illustrates the Root Mean Squared Error (RMSE) value.

(15) $${\;R}^{2}=\frac{\sum_{i=1}^{n}{\left(\theta_{i}-\bar{\theta }\right)}^{2}-\sum_{i=1}^{n}{\left(\theta_{i}-{\tilde{\theta }}_{\left(i\right)}\right)}^{2}}{\sum_{i=1}^{n}{\left(\theta_{i}-\bar{\theta }\right)}^{2}}$$

(16) $$MSE=\frac{1}{n}\sum_{i=1}^{n}{\left(\theta_{i}-{\tilde{\theta }}_{\left(i\right)}\right)}^{2}$$

(17) $$RMSE=\sqrt{\frac{1}{n}\sum_{i=1}^{n}{\left(\theta_{i}-{\tilde{\theta }}_{\left(i\right)}\right)}^{2}\;}$$

where, $\theta_{i}$ and ${\tilde{\theta }}_{\left(i\right)}$ represent the measured and estimated temperatures, $\bar{\theta }$ denotes the mean value of the measured temperature, n is sample size.

## 4 Case study and results

This section expounds on the expected outcome from the established ANFIS model to estimate the transformer top oil and hot spot temperature of a selected 60/90MVA power transformer. The ANFIS model's performance was compared with some chosen existing thermal models.

### 4.1 Configuration of the case study transformer

A three-phase power transformer with a capacity 60/90MVA under Oil Natural Air Natural/Oil Natural Air Forced (ONAN/ONAF) cooling mode was utilized for the purpose of data collection for this study. An hourly sampling rate was used to obtain online data of top oil temperature, winding temperature, loading and ambient temperatures from a monitoring system. An assumption is made in this study that the collected data used for training the model was accurate to a level of 95% confidence interval to take care of some processing and instrument inaccuracies. Table 1 highlights a summary of the specifications and thermal model characteristics of the transformer.

**Table 1:** Variables for thermal modelling.

Parameter	Description
Cooling Mode	ONAN/ONAF
Rated Power (MVA)	60/90
Rated Voltage (kV)	132/33
Rated Current (A)	393.6/1574.6
*R*	6.5
${\Delta \theta }_{o.R}$	47.3 °C
${\Delta \theta }_{hs,R}$	20.3 °C
$\tau_{o,R}$ (mins)	108
$\tau_{wd,R}$ (mins)	6
Total number of fans	10
Frequency	50 Hz
Manufacture Year	2016

### 4.2 Results and discussions

**Fig. 8:**
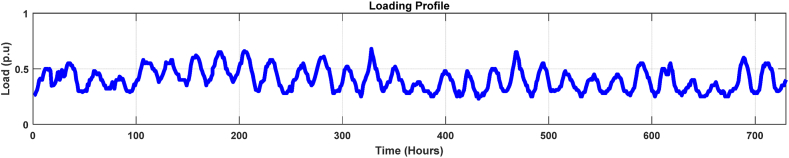
Loading profile in the off-peak load season.

**Fig. 9:**
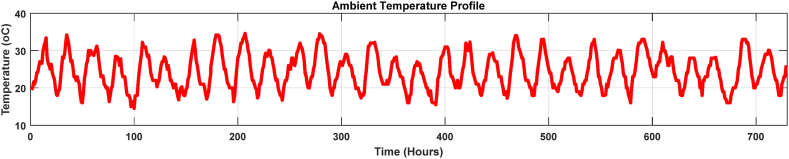
Ambient temperature profile.

Fig. 8, Fig. 9 display the power transformer's three-month loading profile and ambient temperature. Fig. 8 shows that the transformer delivery is below the 50% mark of its ultimate capacity, and the load conditions change regularly. This signifies that the transformer was operating under light loading period. It is acknowledged that there is a second unit running in the same substation to split the load. This had the dual purpose of lowering thermal stress (high temperatures) and extending the lifespan of both transformers and also allow continuity of power flow during faults or maintenance of the other unit.

**Fig. 10:**
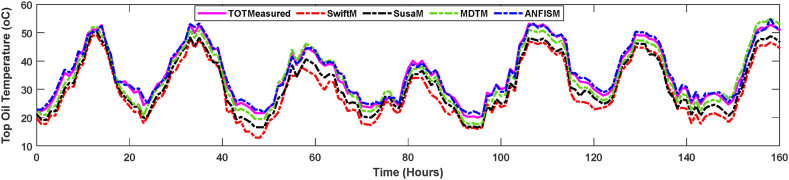
Comparison between modelled and measured top oil temperature of the case study transformer.

**Fig. 11:**
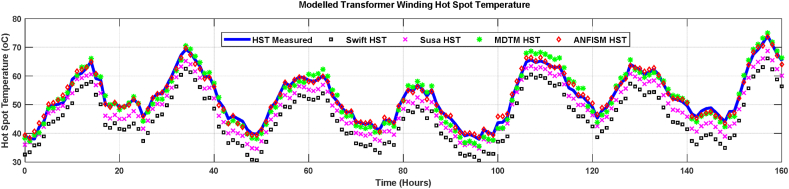
Measured and Modelled Hot spot temperatures.

Fig. 10 demonstrates comparisons between various thermal model output and field measurements of top oil temperatures for the case study transformer. Projected hot spot temperatures from the various models, including measured hot spot temperatures, are shown in Fig. 11. Comparisons include Swift [31], Susa [19], and Moisture-Dependent Thermal Model (MDTM) [20]. The proposed thermal models' simulated results (Fig. 10, Fig. 11) concur with the values that have been measured. It is observed that MDTM was also quite similar to the field recorded data since they considered the impact of moisture in computing the non-linear thermal resistance of transformer insulation. The developed ANFIS based model managed to track well the temperature profile of the measured field reading as it takes advantage of adaptive learning and mapping of inputs into projected output. Thus, the proposed model outperforms the other models.

**Fig. 12:**
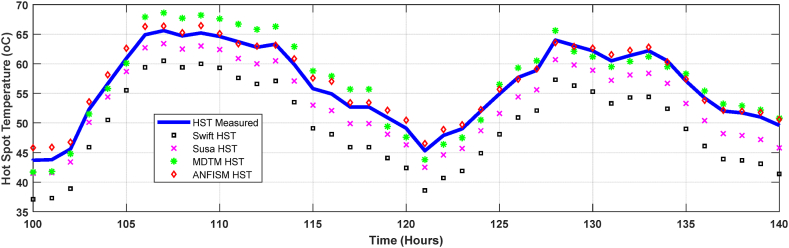
Magnified modelled and measured hot spot temperature.

A magnified view of the modelled and observed hot spot and top oil temperatures from 100 to 140 h in Fig. 11 is depicted in Fig. 12. This enabled a better analogy of hot spot temperatures determined by various models. Compared to measured top oil temperatures, calculated average percentage errors for Swift, Susa, MTDM and proposed ANFIS models are 7.4%, 5.2%, 3.8% and 2.1%, respectively. Swift's models exhibit seemingly large TOT and HST estimation errors of 7.4% and 6.6%, as illustrated in Fig. 10, Fig. 11, which is consistent with the observations reported in Refs. [16,20,51]. Although, there is a deviation between the measured top oil and hot spot temperatures, the average margin of error of all the modelled temperatures are within the allowable industrial error range, illustrating the adequacy of all the models compared in this paper.

**Fig. 13:**
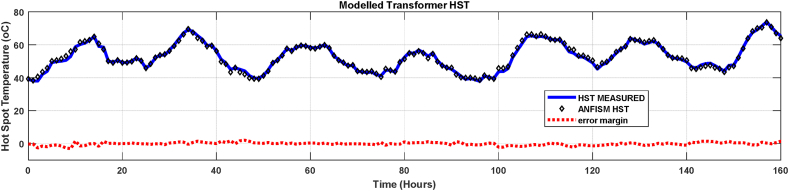
ANFIS Model vs Measured HST.

The comparison curve between the HST measured, the modelled ANFIS values and the prediction error margin of the 160-h group test samples are shown in Fig. 13. The maximum temperature difference of test samples of the three months hourly data-set with ANFIS method is 7.3 °C. Due to its ability to learn and adjust the membership function during training of the data set, the ANFIS model managed to track well the measured temperature profile with an average error margin of 2.1%. Although, the ANFIS model was superior in performance as compared to conventional methods, it still has challenges in the aspect in which the learning is engaged. This is because it has a fixed structure defined during the training step. Thus, other evolving fuzzy models which has characteristics of continuous or incremental learning without demanding a training step can be explored in this area of transformer hot spot temperature prediction. Nevertheless, the ANFIS model can be adopted in the determination of hot spot temperatures in transformer thermal analysis.

The proposed model's performance is evaluated statistically in comparison to other thermal-electric analogy models (Swift, Susa, and Moisture-Dependent Thermal Model (MDTM)) by computing adequacy and accuracy metrics for the projected top oil temperature and hot spot temperatures using equations noted in Section 4.2. Table 2, Table 3 respectively shows the outcome of the performance matrix. The developed ANFIS model achieves the highest coefficient of determination for both TOT and HST (0.98 and 0.96) among the four thermal models, as shown in Table 2. This shows that the ANFIS model is adequate and capable of further projecting top oil and hot spot temperatures. As seen in Table 3, the established ANFIS has the lowest mean squared error. This is another strength that the ANFIS model can consistently estimate the transformer TOT and HST with the highest degree of accuracy as compared to the three models. The three conventional thermal models' reliance on intuitive parameter determination could have led to some degree of uncertainty. In turn, it will consequently result in some errors in the estimation of hot spot temperature. Additionally, the ANFIS based model was strictly optimized for this specific unit, thanks to the learning process that used comprehensive real data from a notable time frame period, hence, superior results.

**Table 2:** Suitability Metrics of the models.

R^2^	Swift	Susa	MDTM	ANFISM
*TOT*	0.77	0.86	0.92	0.98
*HST*	0.73	0.83	0.94	0.96

**Table 3:** Accuracy Metrics of the models.

MSE	Swift	Susa	MDTM	ANFISM
**TOT**	54.7	30.6	13.4	7.8
**HST**	43.5	22.1	15.8	10.3

The conventional models, on the other hand, are distinctive in that they normally perform better for full-load (or even over-load) applications than for units with low loads. A key concern is the accuracy of the data from the heat-run test. Since measured data are reliable during factory tests, they could drastically differ during actual use, which could lead to glaring inaccuracies in temperature modeling utilizing those parameters. Thus, all of its inputs, including those from the heat-run tests, should be evaluated in the appropriate time steps in order to reduce the error introduced by the conventional models.

As confirmed by the performance matrix, the established ANFIS technique has established more accurate top oil and hot spot temperatures compared to the current Swift, Susa and MDTM methodologies. Though the thermal models that are in existence can estimate and track the thermal behavior of power transformers, it was observed that the proposed and the other models can be further extended to determine the transformer thermal temperatures of in-service transformers by fully determining variables not limited to the thermal parameters, measurement of oil temperature and moisture motion, the acquisition of dynamic loading trends, tap changing and environmental temperature among other parameters.

## 5 Conclusions

In this study, a framework based on the ANFIS algorithm for the dynamic thermal behavior of in-service power transformers has been proposed. The suggested approach has been simulated and verified using a case study of a low-loaded mineral oil filled substation power transformer unit. The simulation results demonstrated that using ANFIS' explicit learning capability from the expert knowledge dataset to improve its performance prediction capabilities led to a hot spot temperature determination that was more accurate than those produced by the traditional Swift model, Swift's model, and Moisture Dependent Thermal model.

Although the approach considered in this study demonstrated high accuracy of TOT and HST prediction, temperature by itself cannot provide explicitly essential information about the transformers' general technical state. Consequently, it ought to be considered as one of the many variables that are examined collectively to complete the transformer's technical state assessment aiding in asset management. The quality of the learning data set is a major issue that the suggested approach can further address because it is very susceptible to the use of inaccurate or incomplete data. Therefore, there is need to involve data processing and cleaning techniques before subjecting that data for training and testing purposes. Also, another area of improvement is incorporating more variables like solar radiation, wind speed, and water content (moisture) if the data is available. This can also improve the accuracy of utilizing soft computing techniques in transformer thermal analysis models. Nevertheless, the proposed scheme can aid asset managers in predicting correctly the thermal behavior of a mineral oil-filled power transformer.

## Data availability

Data used in this paper can be accessible upon request.

## CRediT authorship contribution statement

**Edwell T. Mharakurwa:** Writing – review & editing, Writing – original draft, Validation, Methodology, Formal analysis, Data curation, Conceptualization. **Dorothy W. Gicheru:** Writing – review & editing, Data curation.

## Declaration of competing interest

The authors declare that they have no known competing financial interests or personal relationships that could have appeared to influence the work reported in this paper.
